# Protective and Detoxifying Effects Conferred by Dietary Selenium and Curcumin against AFB1-Mediated Toxicity in Livestock: A Review

**DOI:** 10.3390/toxins10010025

**Published:** 2018-01-02

**Authors:** Aniket Limaye, Roch-Chui Yu, Cheng-Chun Chou, Je-Ruei Liu, Kuan-Chen Cheng

**Affiliations:** 1 Institute of Biotechnology, National Taiwan University, Taipei 10617, Taiwan; d01642014@ntu.edu.tw; 2 Graduate Institute of Food Science Technology, National Taiwan University, Taipei 10617, Taiwan; yurc@ntu.edu.tw; 3 Department of Medical Research, China Medical University Hospital, China Medical University, Taichung 404, Taiwan; fstcchou@ntu.edu.tw

**Keywords:** aflatoxin B1, anti-AFB1 dietary supplement, curcumin, selenium, antioxidant status, We have reviewed protective roles of selenium and curcumin in specific relation to tacking AFB1 toxicity in the livestock along with their reported underlying mechanisms for the potential feed applications catering across a broad host range.

## Abstract

Aflatoxin B1 (AFB1), among other aflatoxins of the aflatoxin family, is the most carcinogenic and hazardous mycotoxin to animals and human beings with very high potency leading to aflatoxicosis. Selenium is an essential trace mineral possessing powerful antioxidant functions. Selenium is widely reported as an effective antioxidant against aflatoxicosis. By preventing oxidative liver damage, suppressing pro-apoptotic proteins and improving immune status in AFB1 affected animals; selenium confers specific protection against AFB1 toxicity. Meticulous supplementation of animal feed by elemental selenium in the organic and inorganic forms has proven to be effective to ameliorate AFB1 toxicity. Curcumin is another dietary agent of importance in tackling aflatoxicosis. Curcumin is one of the major active ingredients in the tubers of a spice *Curcuma longa* L., a widely reported antioxidant, anticarcinogenic agent with reported protective potential against aflatoxin-mediated liver damage. Curcumin restricts the aflatoxigenic potential of *Aspergillus* *flavus.* Curcumin inhibits cytochrome P450 isoenzymes, particularly CYP2A6 isoform; thereby reducing the formation of AFB1-8, 9-epoxide and other toxic metabolites causing aflatoxicosis. In this review, we have briefly reviewed important aflatoxicosis symptoms among animals. With the main focus on curcumin and selenium, we have reviewed their underlying protective mechanisms in different animals along with their extraction and production methods for feed applications.

## 1. General Introduction: Aflatoxins, Aflatoxin B1, and Aflatoxicosis

Aflatoxins constitute a family of secondary metabolites mainly produced by *Aspergillus flavus* and *Aspergillus parasiticus* [1]. Aflatoxins are known to affect important agricultural feed and food commodities including dried fruits, oilseed meals, spices, and cereals. Aflatoxins are difuranocoumarin derivatives comprising aflatoxin B1, B2, G1, G2, M1, and M2 wherein AFB1 shows higher toxicity with most frequent encounters [2]. The different isomers of aflatoxins are identified according to the fluorescence color; for example, B stands for blue and G for green. *Aspergillus flavus* produces AFB1 and AFB2*. Aspergillus parasiticus* produces AFB1, AFG1, and AFG2. Aflatoxin M1 is 4-hydroxylated metabolized form of AFB1 found in human breast milk and bovine milk [3]. International Agency for Research on Cancer (IARC), has categorized naturally occurring aflatoxins as group 1 agents, being carcinogenic to the human beings. AFB1 is a reported hepatocarcinogen [4]. Aflatoxin prevalence in animal feed is significant in most of the developing countries located in the geographical region between 35° N and 35° S of latitude [5]. AFB1 affected geographical zone is increasing with new territory in South Eastern Europe due to increased average temperature [6]. The aflatoxigenic *Aspergilli* grow in humid environments which can be found in the storage areas of harvested food material. The secretion of AFB1 depends on abiotic factors including humidity, temperature, presence of oxidative stress, growth substrate, storage material, and sunlight [7]. The genetic mechanism of stress-induced AFB1 secretion is well documented. The biosynthetic cluster of aflatoxin production consists of 25 genes closely located at the telomere of the third chromosome flanked by four putative sugar utilization genes [8]. In the case of *Aspergillus parasiticus*, oxidative stress triggers at least four regulatory transcription factors (AtfB, SrrA, AP-1, and MsnA) which induce aflatoxin biosynthesis [9].

AFB1 toxicity in livestock and human beings is a major economic and health concern. Approximately 500 million human beings, mostly belonging to poor communities from countries located in sub-Saharan Africa, Latin America, and Asia, are exposed continuously to aflatoxin levels higher than international norms [10]. Dietary exposure of AFB1 through infected food ingredients has dose-dependent severe implications on livestock and humans. Upon metabolic activation of AFB1, predominantly in the hepatocytes by cytochrome 450 (CYP450) enzymes, toxic AFB1-8, 9-epoxide is formed which covalently binds to nucleotides and proteins [11]. CYP2A13, a predominantly human respiratory tract enzyme, has been shown to be responsible for the bioconversion of AFB1 to its AFB1-8, 9-epoxide [12]. Aflatoxicosis results from dietary ingestion of aflatoxin that may lead to the acute condition of liver cirrhosis and death or nutritional and immunological consequences depending upon the dosage [1]. AFB1 toxicity in different species varies as per the age, gender, species-specific AFB1 sensitivity, dosage, and duration of toxin exposure [13]. AFB1 causes immunosuppression, carcinogenicity, teratogenicity, and mutagenicity [14].

Aflatoxicosis in chicken is generally characterized by weakness, decrease in weight gain, feed efficiency, and egg production to more severe symptoms involving hemorrhagic liver, elevated lipid levels [15]. In poultry, the immune system is severely affected by AFB1 resulting in reduced subsets of T cell population, decreased mRNA expression of cytokines in the small intestine [16]. Peng et al. [17] have shown that AFB1 dietary exposure in the broilers results in the immunosuppression by immune organ lesions due to increased apoptotic cells and impaired membrane systems. Apart from the physiological effects, AFB1 is also carried over into the chicken eggs [18].

AFB1 contaminated feed in swine creates similar symptoms besides poultry. The symptoms involve reduced feed intake and body weight gain, impaired liver and immune functions, as well as altered serum biochemical parameters [19]. Aflatoxicosis in swine leads to an increase in the weight of kidney, liver, spleen, and pancreas, and an increase in liver-specific enzymes. Along with physiological effects, the immune system is severely affected by aflatoxicosis [20,21]. Dietary AFB1 exposure in swine results in impaired lymphocyte activation, negative influence on vaccine efficiency and delayed cell-mediated immune response. It leads to the heightened expression of pro-inflammatory cytokines IL-6, IFN-γ, and IL-10 [22].

Dairy cattle are relatively more resistant to mycotoxins due to the ability of rumen microflora to biotransform the toxins into less toxic or non-toxic metabolites upon ingestion [23]. However, AFB1 can directly affect rumen microflora metabolism, consequently hampering ruminal digestive capacity [24]. Various cattle health symptoms including the birth of small and unhealthy calves, diarrhea, acute mastitis, respiratory problems, rectal prolapse, and hair loss have been reported [25]. AFB1 toxicity in cattle is of dual significance as it affects the animal growth performance, as well as it leads to milk carryover as AFM1 in the case of lactating cows. Although the toxicity of AFM1 is lower than AFB1, AFM1 can cause DNA damage, gene mutation, chromosomal aberrations, and cell transformation in mammalians cells in vitro, in insects, lower eukaryotes and bacteria [26].

While taking an in-depth account of species-wide AFB1 toxicity is always vital to understand broader implications of AFB1 toxicity, we will focus our discussion on aflatoxicosis prevention by dietary supplements. Moreover, there are numerous anti-AFB1 agents reported, but the application ease and scalability of many of the reported candidates has been questionable. Most of the herbal extracts, essential oils with notable anti-AFB1 activity are commercially non-viable due to limitations in the raw material as quantity, consistent quality, processing complexities and limited final yields. For many of the reported anti-AFB1 additives, there are no comprehensive study reports covering a broad range of animal hosts. For an effective anti-AFB1 agent, it is critical to have better feed safety, high bioavailability, protective hepatic, and immune functions, as well as feasible extracting methods for commercial manufacture. In this review, we will attempt to elaborate on protective effects of selenium and curcumin which fit criteria for the effective anti-AFB1 dietary agents. In the forthcoming sections, we will review the two dietary feed supplements; selenium and curcumin in specific relation to AFB1 toxicity alleviation along with important considerations in their application and production.

## 2. Anti-AFB1 Dietary Agents

The dietary agents consist of various essential oils, probiotic bacteria, extracts of herbal and microbial origins, mannan-oligosaccharides, and chemical agents. It is essential to note numerous reports on anti-AFB1 dietary sources over the decades, underscoring the severity of AFB1 toxicity. In Table 1, we have attempted to enlist reported dietary agents representatively. This table selectively highlights important reports on the dietary application of anti-AFB1 agents to prevent aflatoxicosis in animals. We have broadly differentiated them into two categories according to their origin as chemical and biological agents. Biological agents are further classified as plant origin and microbial origin agents.

## 3. Selenium and Curcumin: General Introduction and Protective Mechanism

Due to an ever-growing plethora of dietary compounds with anti-AFB1 potential, it is challenging to identify practicable agents with better efficacy in tackling AFB1 toxicity. Of various reports, considerable attention has been given to protective effects of selenium and curcumin recently. In this review, we will focus on the application and mechanism of two important anti-AFB1 dietary agents, namely, selenium and curcumin. Selenium is previously demonstrated to be AFB1 detoxifying with immunoprotective potential while curcumin has proven to be an aflatoxicosis mitigating agent [64]. Although there are many reports on aflatoxicosis prevention in different animal species, these findings are needed to be reviewed comprehensively.

This review discusses protective effects of selenium and curcumin supplementations in poultry, duck, swine, and rats, addressing reported protective mechanisms. Before venturing into describing the actual anti-AFB1 roles of these dietary supplements, it is beneficial to review their basic chemistry and underlying protective mechanisms. In the following sections, we will review the chemistry, general mode of action; specific anti-AFB1 mechanisms in different animals, as well as selenium and curcumin feed supplementation strategies.

### 3.1. Selenium: Immunoprotective Antioxidant and Essential Trace Mineral

**Figure 1:**
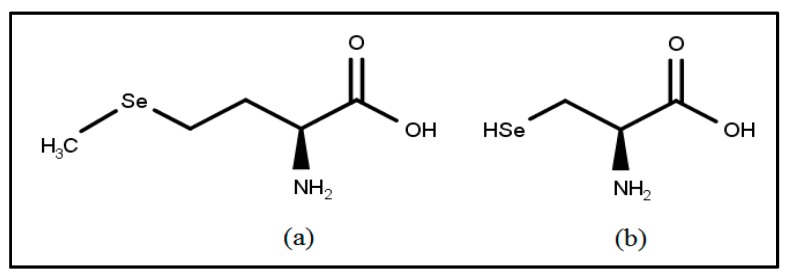
Chemical structures of (**a**) selenomethionine and (**b**) selenocysteine.

Selenium is an element discovered by Berzelius in 1817. It is a rare, but widespread, element with an abundance of 0.05–0.09 parts per million [65]. It is similar to phosphorus in chemical properties. Selenium occurs in organic, as well as inorganic, forms. It is present in the inorganic form as selenites and selenates. Selenoamino acids, selenopeptides, and selenoproteins are the organic forms of selenium. Selenols are one of the main classes of organoselenium compounds with amino acid selenocysteine as the most important selenol [66] (Figure 1).

#### Selenium: General Mechanism of Action

Organic selenoproteins were discovered in the year 1847 [67]. Selenomethionine is an important dietary source of selenium (Figure 1). Inorganic selenium is converted by plants into selenomethionine. Selenomethionine gets converted to selenocysteine in the animals by transsulfuration pathway. Selenocysteine gets further transformed into selenophosphate by selenophosphate synthase [68]. The specific biochemical role of selenium as the functional component of mammalian antioxidant enzyme glutathione peroxidase (GPx) has been demonstrated [69]. The ping-pong mechanism of GPx-mediated peroxidase action is elaborated in detail [70]. The selenolate (GPx-Se-) form of selenocysteine carries out redox reactions. Selenolate (GPx-Se-) is oxidized to selenenic acid (GPxSeOH) to reduce the peroxide (ROOH). The enzyme is regenerated by two molecules of glutathione (GSH). GSH forms a selenenylsulfide (GPx-Se-SG) with the release of a water molecule. GSH regenerates selenolate by the formation of GSSG in the next step [70]. The importance of selenium in various organ functioning is well documented [71,72,73]. Due to its broad implications on mammalian health, the specific application and protective mechanisms of selenium in context to AFB1 will be discussed in upcoming sections.

### 3.2. Curcumin: A Hepatoprotective Phytochemical

AFB1, upon enzymatic activation, is shown to impart oxidative stress to the living cells. Hence, substances with antioxidant properties have proven to be effective to ameliorate AFB1 induced oxidative stress. Phytochemicals are highly sought after targets to treat stress-induced tissue abnormalities. Turmeric is a household spice with medicinal properties. It is used in the Indian subcontinent and Southeast Asia. It is also used widely in Indian, as well as in Chinese traditional medicine since antiquity. Chemically, *Curcuma longa* L. is composed of water-insoluble curcuminoids (including curcumin), a water-soluble peptide called turmerin, along with turmerones, altertones, zingiberene as essential oils [74] . Depending on the region and soil conditions, curcuminoids constitute approximately 2–9% of turmeric by dry weight, a group of compounds comprising of curumin, demethoxycurcumin, bis-demthoxycurcumin, and cyclic curcumin. Curcumin was first isolated from turmeric in 1815. Curcumin (1E,6E)-1,7-bis(4-hydroxy-3-methoxyphenyl)-1,6-heptadiene-3,5-dione is the major component of curcuminoid extract obtained from the turmeric rhizomes [75]. Curcumin makes up to 60–70% of the curcuminoid crude extract by weight [76]. Curcumin is one of the most extensively studied phytochemicals concerning chemopreventive potential. The potential chemopreventive role of curcumin in cancer prevention has been reported in a mouse skin model [77], and in epithelial colon cells [78] by inhibition of cyclo-oxygenase 2 (COX2) expression through inhibition of κB degradation by downregulation of NF-κB-inducing kinase (NIK) and IκB kinase (IKK) α/β. Apart from the antioxidant activity, curcumin possesses a strong cytoprotective potential for liver protection [79] and potential as an anti-breast cancer agent [80].

#### Curcumin: General Mechanism of Action

**Figure 2:**
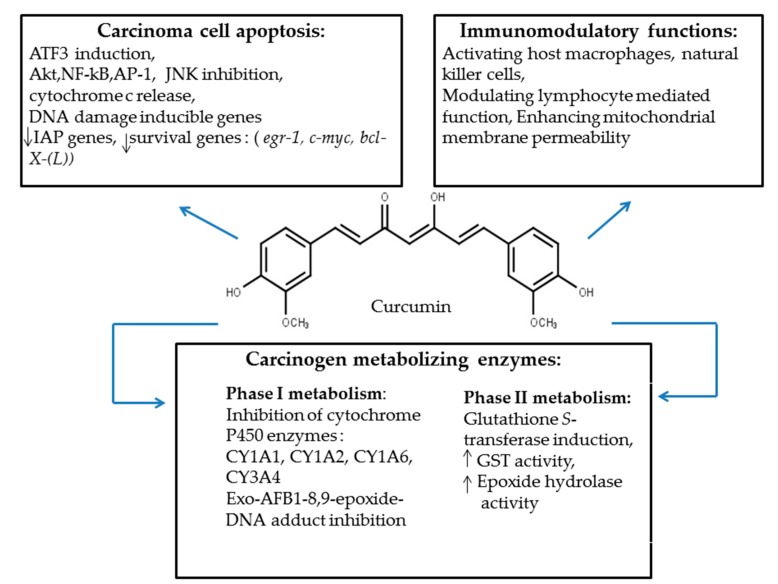
Overview of curcumin protective functions.

There are multiple protective functions rendered by curcumin in different host species [81,82,83]. Figure 2 describes medicinal properties of curcumin which have a protective roles against AFB1-mediated toxicity.

## 4. Selenium-Mediated AFB1 Toxicity Control and Protective Mechanisms

Ameliorative evidence of selenium in the animal diet to mitigate AFB1-mediated toxicity has been reported over the years in different animal species [84,85,86,87,88]. The protective role of selenium has been studied over a longer time span. However, the underlying biochemical and immunoprotective mechanisms have been studied more recently with the main focus on chickens and ducks. Selenium supplementation research to counter AFB1 toxicity in swine is still in the early developmental stage.

### 4.1. Selenium-Mediated Protection

The ability of selenium in mitigating AFB1 induced immunosuppression, liver dysfunction and the apoptotic damage is central in conferring anti-AFB1 protection through feed supplementation. Due to the prevalence of AFB1 in animal feed and its diverse host range, protective mechanisms underscoring the role of dietary selenium in toxicity alleviation have been reported in many animals including swine, poultry, and ducks. Reported anti-AFB1 protective studies revealing biochemical and molecular evidence provide insight into the role of selenium in the protection of liver, spleen, and thymus. We will review selenium-mediated anti-AFB1 protection in more detail in the following sections.

#### 4.1.1. Selenium-Mediated Anti-AFB1 Protection in Ducks

Ducks, especially ducklings, have a very high susceptibility to AFB1 toxicity with severe physio-economic effects [89]. Ducklings have been shown to have improved overall growth performance, improvement in the weight of the bursa of Fabricius, along with significant improvement in IL-2 and IL-6 levels post AFB1 exposure; when supplemented with selenium yeast (SeY) and vitamin E (VE), which is also an effective antioxidant. This study implemented diets containing either 0% contaminated corn or 100% AFB1 contaminated corn with or without VE and SeY. Fifty IU of vitamin E and 0.2 mg SeY/Kg of body weight were administered. Levels of AFB1 in the starter diets were 130.5 and 142.4 μg/kg, while grower diet contained 152.6 and 157.1 μg/kg with or without VE and SeY, respectively [90]. Another study has reported similar ameliorative effects in case of sodium selenite supplementation (1 mg/kg body weight) with intragastric administration of AFB1 (0.1 mg/kg body weight) significantly improving the bursa of Fabricius indices, thymus indices, and body weight indices compared with ducklings fed with non Se-supplemented AFB1 contaminated feed [87].

Liver dysfunction, being one of the hallmarks of aflatoxicosis, is a major target to understand the underlying protective molecular mechanism mediated by selenium. The mitochondria-dependent apoptotic pathway involves mitochondrial outer membrane permeabilization, which eventually leads to apoptosis by activation of caspase-3. A significant decrease in serum alanine transferase (ALT) and aspartate transaminase activities (AST), the diagnostic evaluation markers of hepatic injury which were significantly heightened in AFB1 exposure group, is reported [91]. Selenium treatment of 1 mg/kg body weight of ducklings resulted in significant reduction in the serum levels of these hepatodamage marker enzymes. The same article demonstrated an important anti-apoptotic function of selenium. The selenium-supplemented AFB1 group, in contrast with the non-supplemented AFB1group, with a 0.1 mg/kg body weight AFB1 dosage, showed a significant decrease in Bcl-2/Bax expression ratio. Bcl-2 is an anti-apoptotic protein, Bax being a pro-apoptotic protein. Selenium also led to significant reduction in caspase-3 and p53 levels after the treatment of 21 days. This study showed that selenium-mediated AFB1 protection involved protecting liver dysfunction by selectively promoting pro-apoptotic protein repression.

Hepatic AFB1 metabolism is known to induce intracellular reactive oxygen metabolites. Selenium supplementation in this case significantly reduced the expression of hepatic mitochondrial levels of superoxide dismutase (SOD), catalase (CAT), glutathione peroxidase (GSH-Px), and glutathione reductase (GR) [92]. Selenium plays an important antioxidant role conferring hepatic protection. In the year 2015, the same research group has evaluated hepatic mitochondrial oxidative DNA damage development and its mitigation by selenium supplementation. They have reported nine SNPs in the mitochondrial DNA (mtDNA) D-loop region found frequently in the AFB1 treatment group (0.1 mg/kg) compared to AFB1-Se supplemented (1 mg/kg) treatment group. This region is a non-encoding region interfering in the transcription of the entire mtDNA genome. Selenium was demonstrated to play an antioxidant role, reducing free radical accumulation and, hence, conferring liver protection by extenuating hepato-morphological changes, mitochondrial swelling, and mtDNA damage [93].

#### 4.1.2. Selenium-Mediated Anti-AFB1 Protection in Poultry

As the most important avian species in animal husbandry and having high susceptibility towards aflatoxicosis, there have been studies reporting protective effects of selenium in chickens [94,95,96,97,98].

Coherent with the histopathological damage caused in ducks, AFB1 causes liver damage in poultry. AFB1 is shown to target the bursa of Fabricius and the cytotoxic arm of the immune system. The bursa of Fabricius is an organ of proliferation and diversification of B cells. AFB1 exposure is shown to reduce its weight along with excess apoptosis and pathological lesions. Selenium supplementation improved the relative weight of the AFB-affected bursa of Fabricius. Similar to the case with ducklings, AFB1 is shown to be up-regulating the hepatic mitochondrial apoptotic proteins, caspase-3 and Bax, as well as reducing the concentration of immunoglobulins IgA, IgG, and IgM. Selenium supplementation in the broilers exposed to 0.3 mg/kg AFB1 displayed a reduction in Bax, increase in Bcl-2 expression competing with Bax to inhibit apoptosis along with improving immunoglobulins, thereby restoring humoral immune function. This study tested three dosages of selenium supplementation namely; 0.2, 0.4, and 0.6 mg/kg, of which the 0.4 mg/kg dosage rendered optimal protection against 0.3 mg/kg AFB1 [94].

AFB1 induced mucosal immunocompromising, and its protective reversal by selenium supplementation with molecular evidence has been reported. AFB1 damages cellular immunity in the ileum by downregulating matured T cells. The thymus is a central immune organ involved in the transgression of matured T cells. AFB1 showed significant relative weight reduction of chicken thymus, which was countered by selenium supplementation. IL-2 and IL-6 are crucial interleukins acting as a T cell growth promoter and proliferator, respectively. TNF-α, on the other hand, is functional in the intestinal permeability. Activated T lymphocytes secrete these cytokines. 0.3 mg/kg addition of AFB1 altered their protein expressions significantly, as verified by mRNA estimations. This study has demonstrated that AFB1 feed addition caused the reduction in cellular immune function of ilium through downregulating T-cell subsets. Percentages of CD3^+^, CD3^+^ CD4^+^, CD3^+^, and CD8^+^ T cells in intraepithelial lymphocytes (IELs) and lamina propria lymphocytes (LPL) were significantly reduced; the CD4^+^/CD8^+^ ratio in IELs was decreased compared with the control group (basal diet). However, supplementing the AFB1 contaminated feed with 0.4 mg/kg dietary selenium (sodium selenite) restored the T-cell subtype populations close to those in the control group. Selenium supplementation reversed the protein suppression with a significant increase in the mRNA expressions of IL-2, IL-6, and TNF-α, and a corresponding increase in T-cell subset populations thereby improving the cellular immunity of ileum [99]. This report also showed an important finding that excess of selenium in chicken diet resulted in adverse immune effects with weakened T cell immunity.

AFB1 is a planer molecule being inert without toxicity before being activated structurally by enzymatic or other means. AFB1 in hepatocytes gets converted into exo-AFB1-8, 9-epoxide by cytochrome P450 (CYP450) family oxidase enzymes. This electrophilic metabolite is unstable and very toxic as it gets directly intercalated into DNA and forms N7 guanine adducts leading to mutations [100,101]. Bioactivation of AFB1 is a crucial event in aflatoxicosis manifestation. As a result, it is important to find and understand regulatory role of selenium in controlling CYP450 enzymes. According to a recent report, four major enzymes namely CYP1A1, CYP1A2, CYP2A6, and CYP3A4 are selectively upregulated, leading to AFB1 toxicity causing the liver damage [88]. Interestingly, selenium inclusion in the feed diet resulted in prevention of upregulation of these CYP enzymes with no signs of liver damage and significantly reduced AFB1-DNA adducts formation. The basal diets provided 0.10 mg/kg Se, while additional selenium was supplemented with sodium selenite to the final concentration of 0.2 and 0.5 mg/kg of the experimental diets, and 100 µg/kg AFB1 dose. The same study also gives preliminary insights about the involvement of specific selenoprotein genes in AFB1 detoxification in chicken. Six out of 26 tested chicken selenoprotein genes were upregulated, namely, *Gpx3*, *Txnrd1*, *Txnrd2*, *Txnrd3*, *Dio2*, and *Sepn1*. This study reports a strong correlation between the hepatic *Gpx3*, *Txnrd1*, *Txnrd2*, and *Txnrd3* mRNA levels with GPX and TXNRD enzyme activities. Although the role of *Dio2* in AFB1 detoxification is unknown, the rest of the upregulated genes code for the proteins with antioxidant activity. Further validation studies are essential to confirm the correlation between upregulated mRNA levels of the antioxidant genes and their corresponding enzymatic activities, as mRNA may undergo selective translation as well.

Supplementation of primary chicken hepatocytes with 2 µM selenomethionine (SeMet) showed an increase in expression levels of SelW mRNA. SelW is a selenoprotein with an essential role in maintaining normal liver function. According to this recent report, SeMet suppressed the expression of CYP450 1A5 enzyme which activates AFB1 to transform to toxic AFBO form; while SelW knockdown with SelW-specific siRNA significantly increased mRNA and protein levels of CYP450 1A5, thus indicating the protective role of SeMet [102].

#### 4.1.3. Selenium-Mediated Anti-AFB1 Protection in Swine

Unlike poultry or ducks, the selenium-mediated protection post AFB1 exposure is not reported in depth so far. To the best of our knowledge, there is still limited information available about the general protective effects conferred by selenium in swine [86]. According to the report, Se administered in combination with warfarin showed to confer protection against AFB1. The protective mechanism has been elucidated in vitro in porcine splenocytes recently. The study demonstrated selenomethionine (SelMet) protected AFB1 challenged porcine splenocytes by enhancing mRNA and protein expression of glutathione peroxidase 1 (GPx1), selenoprotein S (SelS), and thioredoxin reductase 1 [103]. This study demonstrated SeMet supplementation protected AFB1 challenged splenocytes by restricting GSH depletion. They showed the major function of SelS in AFB1 toxicity alleviation by specifically targeting SelS with SelS siRNA which resulted in impairment of protection with the onset of T-cell immune toxicity. This study showed preliminary evidence of SelS as a major selenoprotein involved in the protective mechanism against AFB1.

## 5. Curcumin-Mediated AFB1 Toxicity Control and Protective Mechanisms

Curcumin, as mentioned in the earlier section is a comprehensively studied antioxidant agent. Many studies have documented the application of curcumin in ameliorating cancer-related damage and carcinogenesis [104,105,106,107,108,109].

Despite the potency and efficacy of curcumin to tackle aflatoxicosis and any other oxidative stress condition, there have been limited reports describing the detailed underlying protective mechanisms. Although there have not been established comprehensive protective mechanisms in different species so far, there is an ever-increasing evidence of curcumin-mediated ameliorative role in aflatoxicosis. Curcumin has been reported to demonstrate protective function in AFB1 inflicted damage to liver, kidneys, and overall growth parameters, mainly in ducks, chickens, and rodents [110,111,112,113,114,115,116,117].

Curcumin helps to maintain the antioxidant status of the cells by scavenging free radicals, inhibiting oxidative enzymes, activating catalases and inducing de novo glutathione synthesis [110]. Most of the research work is focused on the protective effects of curcumin or curcuminoids in rats and poultry. Curcumin protected AFB1 affected mice kidney by significantly increasing contents of ascorbic acid and glutathione contents, decreasing lipid peroxidation with increased catalase and superoxide dismutase activity. In this study, mice were exposed to the low and high dose of AFB1 (750 and 1500 µg/kg body weight) with 2 mg/0.2 mL olive oil/animal/day of pure curcumin [116]. Oral administration of curcumin in rats was shown to restrict the AFB1-mediated oxidative liver damage. It significantly improved antioxidant status of liver by increasing hepatic GSH levels, as well antioxidant enzymes (SOD, CAT, and GSH-Px). Rats were fed with curcumin (200 mg/kg body weight) and resveratrol (10 mg/kg body weight) to study protective effect against aflatoxin B1 (25 μg/kg). Resveratrol (3,4,5′-trihydroxy-trans-stilbene), a natural polyphenol was found to be ineffective in conferring hepatoprotection to mice in contrast to curcumin [110]. Likewise, consistent hepatoprotective action against AFB1 induced damage is reported by upregulation of antioxidant enzymes in rat liver upon oral curcumin administration (15 mg/kg body weight) with an AFB1 dose of (3 mg/kg body weight) [118]. Curcumin is shown to block the transformation of AFB1 to aflatoxicol by competitively inhibiting chicken liver cytosolic AFB1 reductase [119]. Ameliorative effect of total curcuminoids by activating the antioxidant system in the broiler liver damaged by AFB1-mediated oxidation has been reported. In this study, the authors tested increasing concentrations of total curcuminoids (74, 222, 444 mg/kg) with 1 mg/kg dose of AFB1. They found that 222 mg/kg total curcuminoids provided the highest amelioration against AFB1 with a significant increase in lipid peroxide, aqueous peroxide levels and serum biochemical parameters [120]. The protective role of curcumin by improving antioxidant status in the host organs appears to be similar regardless of host species. To obtain a deeper insight into the actual mechanism of the protective role of curcumin, a molecular approach to supplement the conventional and established tests of antioxidant activity measurement and liver histology is required. Important molecular evidence emphasizing curcumin-mediated amelioration against AFB1 is investigated in this study [83]. This study targeted on selective gene expression of hepatic cytochrome P450 isoenzymes which activate AFB1 into carcinogenic AFB1-8, 9-epoxide. They have demonstrated a significant increase in the mRNA levels and/enzyme activities of CYP1A1, CYP1A2, CYP2A6, and CYP3A4 isoenzymes upon AFB1 exposure (100 mg/kg) in broilers which were inhibited in case of curcumin supplementation (150 mg/kg). According to these findings, these isoenzymes are crucial in AFB1 epoxidation in case of mammals as well as aves. Further confirmatory experiments will clearly form a link between curcumin and the CYP450 isoenzymes which could prove effective establishing curcumin as an anti-AFB1 feed additive. Recently, Mohammad et al. [121] have reported new insight into in vivo activation of AFB1 in broilers by the CYP2A6 enzyme. They have reported a dose-dependent hepatoprotection and CYP2A6 activity inhibition. Curcumin supplementation demonstrated a significant reduction in AFB1-DNA adducts formation in contrast to the AFB1 treatment group (5 mg/kg). Supplementation of curcumin with 450 mg/kg feed was found to completely inhibit CYP2A6 enzyme activity. The mRNA expression results demonstrated that curcumin selectively inhibited CYP2A6 expression in case of the AFB1 treatment group with 450 mg/kg curcumin supplementation.

## 6. Selenium Supplementation of Animal Feeds

Interestingly, selenium, unlike other metal cofactors gets cotranslationally incorporated into the polypeptide chain as part of amino acid selenocysteine. Such proteins with selenium as an integral part of the polypeptide chain are known as selenoproteins which are present in eukaryotes, prokaryotes, and archaea. Selenomethionine, an essential amino acid which is not synthesized by higher animals is capable of getting incorporated into the body proteins by methionine replacement [122]. Selenium supplementation to animal feeds is achieved by the inorganic, as well as organic, sources of selenium. Organic selenium in animal feed is in the form of selenomethionine (SeMet), methylselenocystein (MeSeMet), selenium proteinate (Se-proteinate), and selenized yeast (Se-yeast). Inorganic selenium is present in the form of selenite (Se (IV)) or selenate (Se (VI)) (referred to as iSe) [123].

Feed application of inorganic Se is still in practice mainly due to lower cost compared to organic selenium feed additive. However, its application has some significant disadvantages, like the interaction of Se with other minerals and vitamins, low carryover efficiency into milk, eggs, and meat, inability in building Se reserve in the body, resultant excretion due to inefficient gut absorption, and Se reduction due to reducing environment during storage conditions or in the digestive tract [124].

During evolution, all mammals and avian species are adapted to metabolize organic selenium with an active intestinal absorption [125]. Chemical similarity between methionine and SeMet enables its interchangeable role in the cellular process, resulting in building the Se reserves in the muscles, playing a significant role in oxidative stress conditions [126]. It is worth noting to assess the selenium status of the livestock for developing appropriate Se supplementation regime, to control the Se release into the feed. Excess of selenium depending on the source is proven toxic [127,128]. The source of selenium supplementation has to be decided depending on the animal type as there are performance variations depending upon selenium type [129,130,131]. Fairweather-Tait et al. [132] have extensively reviewed selenium bioavailability. The same review has pointed out numerous significant food sources with bioavailable organic selenium which predominantly involve Se-yeast, Brazil nuts, lentils, and enriched wheat.

**Figure 3:**
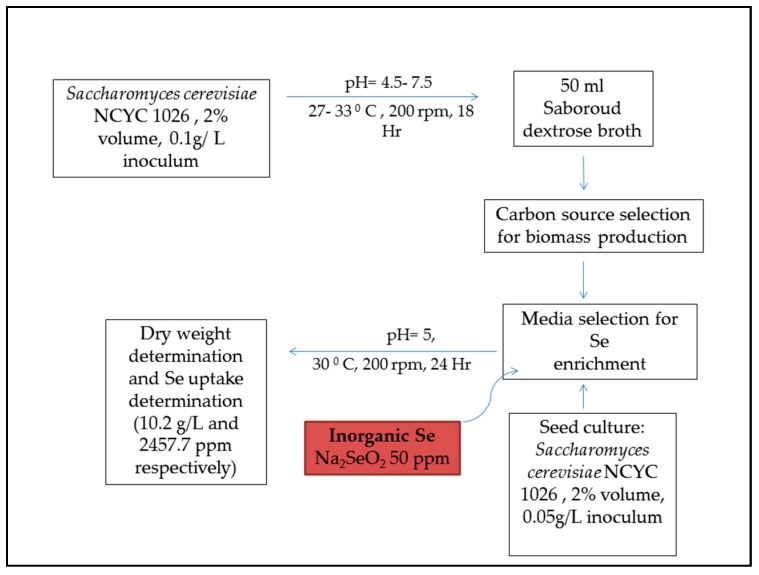
Production of selenium-enriched yeast.

Se-yeast has been an FDA-approved feed supplement since the year 2000 for animal feed. In terms of practicality and efficiency of selenium delivery, Se supplementation in organic form is more efficient in improving growth parameters and antioxidant status of cattle, poultry, and swine [6,124,133]. *Saccharomyces cerevisiae* is used for manufacturing Se enriched yeast [122]. Selenium accumulation in the yeast is a result of the accumulation of various selenoproteins. This complex process depends on the extracellular Se binding on membrane assembly by ligands, as well as intracellular Se accumulation by ion transport. An in-depth account of the accumulation and Se enrichment in the yeast has been reported [134]. The authors have extensively discussed the current information on biotransformation pathways and the effects of different media components on Se metabolism in yeast. Some articles have reported elaborate accounts of yeast-mediated selenium enrichment methods [135,136,137,138]. Figure 3 gives a general scheme of Se yeast production based on Rajashree and Muthukumar, 2013 [137].

## 7. Curcumin Extraction and Feed Application

**Figure 4:**
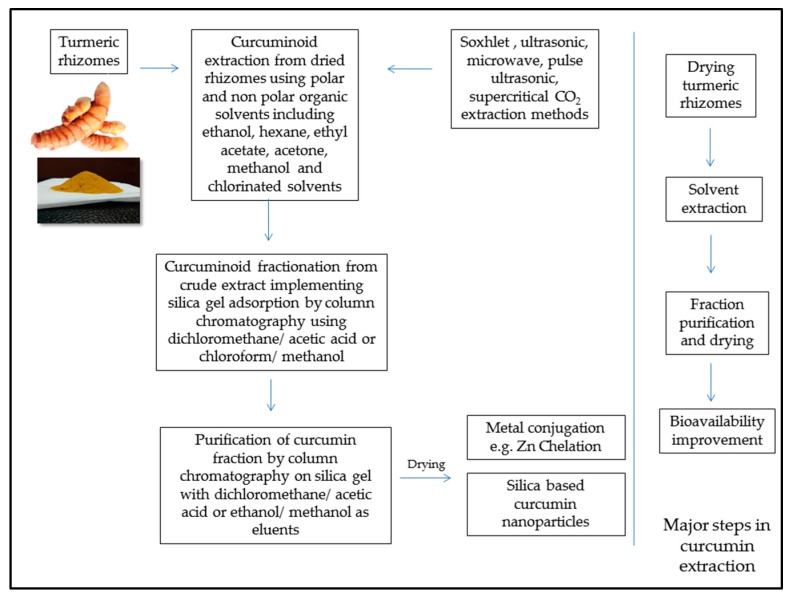
Overview of curcumin extraction and bioavailability improvement approaches.

Curcumin, as discussed earlier has been widely reported to be a beneficial antioxidant agent effective in aflatoxicosis prevention and treatment. Chemistry of curcumin is briefly mentioned in the past section elaborating curcumin as one of the major constituents of curcuminoids which are active components of turmeric rhizomes. Curcumin extraction has been an important research interest in the field of organic chemistry. Priyadarsini (2014) [75] has comprehensively reviewed different extraction methods and curcumin conjugates, along with advances in improvements in curcumin delivery systems involving nanoparticles, due to hydrophobic nature of curcumin. Effective and simple chelation of zinc with curcumin to improve its bioavailability has been demonstrated [139]. Feed addition of curcumin to enhance immune status is well reported as reviewed earlier. However, curcumin application for aflatoxicosis prevention is still in the developing stages with advances in curcumin bioavailability. Figure 4 gives a brief overview of curcumin extraction for potential feed additives.

## 8. Conclusions

There has been constant research progress in the development of effective nutritional feed supplements which can efficiently tackle mycotoxin contamination without compromising nutrient loss. Anti-AFB1 dietary supplements have gathered significant attention due to acute AFB1 toxicity, and also due to the fact that many of these supplements do not pose significant feed safety issues with comparatively easy clearances from feed and drug authorities. However, very few dietary agents with high efficacy against aflatoxicosis have been studied, elaborating underlying molecular mechanism across all important animals of the livestock.

Selenium application in animal feed to ameliorate aflatoxicosis is a growing discipline with promising reports in aves. In case of swine, in vitro results on splenocytes are promising with high potential for in vivo toxin challenge studies. Probiotic selenium as in the form of yeast incorporated organic selenium can prove to be a promising feed supplement in preventing selenium deficiency, along with conferring anti-AFB1 protection. Curcumin has also been demonstrated to be a potent ameliorating agent counteracting AFB1-mediated oxidative liver damage; by selectively targeting CYP450 isoenzymes, thus inhibiting hepatic activation of AFB1 to toxic metabolic forms. More in-depth in vivo studies in animals other than aves will further deepen the insights about protective mechanisms for both of these compounds. With increasing study reports citing protective mechanisms, these two unique nutritional agents hold a great potential to be effective anti-AFB1 supplements with diverse applicability in animal husbandry. In this review, we have attempted to provide a comprehensive, wide-ranging background about protective roles of selenium and curcumin in efficiently mitigating AFB1 toxicity along with essential considerations in their feed applications.

## Figures and Tables

**Table 1:** Table summarizing anti-AFB1 dietary agents in feed applications.

Agent Type	Dietary Agent	Reference
Chemical	Aluminosilicates	[27]
	β-1,3 glucan	[28]
	β-napthoflavone	[29]
	Butylated hydroxianisole	[30]
	Calcium propionate	[31]
	Indole-3-carbinol	[32]
	Oltipraz & Ethoxyquin	[33]
	*N*-acetylcysteine	[34]
	Phenobarbital	[35]
	Triterpenoid	[36]
	Vitamins A, C and E	[37,38,39,40,41]
	Zinc	[42]
Biological	*Azadirachta indica* extract	[43]
(Plant origin)	Chlorophyll	[44]
	Chlorophyllin	[45]
	Coumarin	[46]
	Coffee	[47]
	Essential oils	[48]
	Garlic powders	[49]
	Marine algae extract	[50]
	*Nigella sativa*	[51]
	Silymarin	[52]
	Tea polyphenols	[53]
	Vegetable extracts	[54]
Biological	Atoxigenic strains of *Aspergillus flavus*	[55]
(Microbial origin)	*Bacillus* and *Lactobacillus* spp. probiotics	[56,57,58]
	Mannonoligosaccherides	[59]
	Probiotics mixed with AFB1 degrading enzyme	[60,61]
	Yeast species	[62,63]
